# Blood bank storage of red blood cells increases RBC cytoplasmic membrane order and bending rigidity

**DOI:** 10.1371/journal.pone.0259267

**Published:** 2021-11-12

**Authors:** Sebastian Himbert, Syed M. Qadri, William P. Sheffield, Peter Schubert, Angelo D’Alessandro, Maikel C. Rheinstädter

**Affiliations:** 1 Department of Physics and Astronomy, McMaster University, Hamilton, ON, Canada; 2 Origins Institute, McMaster University, Hamilton, ON, Canada; 3 Faculty of Health Sciences, Ontario Tech University, Oshawa, ON, Canada; 4 Department of Pathology and Molecular Medicine, McMaster University, Hamilton, ON, Canada; 5 Centre for Innovation, Canadian Blood Services, Hamilton, ON, Canada; 6 Centre for Innovation, Canadian Blood Services, Vancouver, BC, Canada; 7 Centre for Blood Research, University of British Columbia, Vancouver, BC, Canada; 8 University of Colorado Denver-Anschutz Medical Campus, Aurora, CO, United States of America; University of Life Sciences in Lublin, POLAND

## Abstract

Blood banks around the world store blood components for several weeks ensuring its availability for transfusion medicine. Red blood cells (RBCs) are known to undergo compositional changes during storage, which may impact the cells’ function and eventually the recipients’ health. We extracted the RBC’s cytoplasmic membrane (RBC_*cm*_) to study the effect of storage on the membranes’ molecular structure and bending rigidity by a combination of X-ray diffraction (XRD), X-ray diffuse scattering (XDS) and coarse grained Molecular Dynamics (MD) simulations. Blood was stored in commercial blood bags for 2 and 5 weeks, respectively and compared to freshly drawn blood. Using mass spectrometry, we measured an increase of fatty acids together with a slight shift towards shorter tail lengths. We observe an increased fraction (6%) of liquid ordered (*l*_*o*_) domains in the RBC_*cm*_s with storage time, and an increased lipid packing in these domains, leading to an increased membrane thickness and membrane order. The size of both, *l*_*o*_ and liquid disordered (*l*_*d*_) lipid domains was found to decrease with increased storage time by up to 25%. XDS experiments reveal a storage dependent increase in the RBC_*cm*_’s bending modulus *κ* by a factor of 2.8, from 1.9 k_B_T to 5.3 k_B_T. MD simulations were conducted in the absence of proteins. The results show that the membrane composition has a small contribution to the increased bending rigidity and suggests additional protein-driven mechanisms.

## Introduction

The long term storage of blood components is essential in transfusion medicine. Blood is first collected from donors and processed into different components, after which they are stored until needed in hospitals around the world. The maximal allowed storage time is 5 to 6 weeks in most countries, depending on the jurisdiction. Red cell concentrate (RC) refers to the concentrated RBC fraction of blood and there is some evidence that the use of older RC in transfusion is accompanied by clinical consequences, such as rapid clearance from the bloodstream of the recipient of membrane-damaged RBCs [1, 2], inflammatory reactions [3], multiple organ dysfunction [4–6] and an increased mortality [5–8]. It is known that RBCs—the most abundant cell type in blood—undergo numerous biochemical, and structural changes during storage, resulting in a decreased resistance against oxidative stress [9–11], damaged membranes [10, 12–14] and reduced deformability [15–19].

The RBCs’ unique ability to deform is intrinsically related to the complex interplay between the spectrin network and the cytoplasmic membrane, which form the outer layer of the cell. When deformations occur on length scales that are smaller than the spacing between the spectrin tethers (≈80 nm), the mechanical properties of the RBC_*cm*_ become dominant [20]. This regime is in particular important for molecular processes, such as the non-active transport of small molecules across the membrane [21].

The storage of RC leads to several changes in the RBC_*cm*_ composition such as protein [11] and lipid [14, 22, 23] oxidation, together with an externalization of charged phosphatidylserine lipids [24]. In addition, changes in the band 3 membrane-protein are associated with the aggregation and binding of hemoglobin to the membrane and an increased removal of stored RBC from the circulation [25]. Controversial information exists on the relative amount of cholesterol in the bilayer. An increase in membrane cholesterol levels during storage has been reported, and speculated to be a result of lipid loss [26, 27], while other articles challenge these findings [28].

Here, we investigated the implications of storage on the molecular structure and bending stiffness of hemoglobin depleted RBC_*cm*_s. RBCs were stored in commercial blood bags for 2 weeks and 5 weeks, respectively, and the RBC_*cm*_s were isolated. We determined the membranes’ thickness, domain sizes and the bending modulus using a combined suite of X-ray diffraction (XRD) and X-ray diffuse scattering (XDS) experiments. We further determined the lipid composition from mass spectrometry and developed coarse grained Molecular Dynamics (MD) models for fresh and stored RBC_*cm*_s. While the structural changes were altogether small, they indicate an increase in molecular packing, thickening and stiffening of RBC_*cm*_s as function of storage time.

## Materials and methods

This research was approved by the Hamilton Integrated Research Ethics Board (HIREB) under approval number 1354-T and by the Canadian Blood Services Research Ethics Board under approval number # 2015.022.

### Preparation of solid supported RBC cytoplasmic membranes

Leukocyte reduced transfusion RCs were provided by the Canadian Blood Services Network Centre for Applied Development (netCAD, Vancouver, BC) and stored in standardized PVC plastic bags in a citrate phosphate dextrose and saline-adenine-glucose-mannitol (CPD-SAGM) solution. The storage bag was stored at 4°C and samples were collected after 2 and 5 weeks, respectively. In addition, fresh blood was collected from volunteers in 10 ml heparinized blood collection tubes. Hemoglobin depleted RBC liposomes were then prepared from all samples following a previously published protocol [29, 30]. Briefly: The whole blood was washed twice and the RBCs were isolated by successive centrifugation and replacing the supernatant with phosphate saline buffer (PBS). The cells were exposed to osmotic stress by mixing hematocrit with lysis buffer (3% PBS buffer, pH 8) at a concentration of 3 vol%. The lysis buffer was pre-chilled to 4°C and the reaction tubes were immediately stored on ice to prevent a fast re-closing of the ruptured cells. ≈ 92% of the initial hemoglobin content were removed through multiple washing steps, as demonstrated in [29]. The protocol results in a white pellet containing empty RBC liposomes. The resulting solution was tip sonicated 20 times for 5 s each at a power of 100 W. The reaction tube was placed on ice during sonication to prevent the sample from overheating. Afterwards, the tube was centrifuged for 15 min at 20,000 g. The supernatant consists of a solution of small, nanometer-sized liposomes at a membrane concentration of ≈14 mg/ml [29].

Multi-lamellar, solid supported membranes were prepared for the X-ray experiments. Membranes were applied onto single-side polished silicon wafers. 100 mm diameter, 300 *μ*m thick silicon wafers were pre-cut into 10 × 10 mm^2^ chips. The wafers were treated with a solution of 15 ml sulfuric acid and 5 ml hydrogen peroxide (Piranha solution) resulting in a hydrophilic surface. This strong oxidizing agent removes all organic contaminants on the surface, but does not disturb the native silicon oxide layer. Each wafer was then thoroughly rinsed with ≈50 ml of ultra pure water (18.2 MΩ⋅cm) and placed on a hot plate (37°C) in a 3-dimensional orbital shaker. 100 *μ*l of the RBC liposome solution was slowly pipetted onto the wafer. The sample was covered with a tilted lid of a petri dish to allow the membrane solution to slowly dry within ≈12 h. The dried wafers were then incubated for 24 h at 97% relative humidity (RH) and 37°C by placing the samples in a sealed container with a saturated K_2_SO_4_ solution. The subsequent drying and incubation of the sample results in a fusion of the RBC liposomes on the silicon surface producing a stack of several hundreds RBC_*cm*_s, which is a prerequisite for the structural X-ray investigations [29].

### X-ray diffraction

X-ray diffraction was performed on a RIGAKU Smartlab diffractometer using a 9 kW (45 kV, 200 mA) CuK*α* rotating anode source with a wavelength of 1.5418 Å and a Rigaku HyPix-3000 2-dimensional semiconductor detector with an area of 3,000 mm^2^ and 100 *μ*m^2^ pixel size. Both source and detector are mounted on movable arms such that the membranes remained horizontal throughout the measurements. The *q*_||_-axis probed the lateral structure, parallel to the wafer surface, and the perpendicular axis, *q*_*z*_, probed out-of-plane structure, perpendicular to the substrate. The focusing multi-layer optics provided a high intensity beam of ≈200 *μ*m with monochromatic X-ray intensities of up to 10^8^ counts/s. The samples were mounted in a custom-built humidity chamber during the experiments. The temperature inside the machine was kept constant at 37°C. Two measurements were performed: perpendicular membrane structure and the electron densities were determined at 88% RH, while the bending rigidity was measured at a high humidity of 99.9% RH.

The membrane orientation *H* was determined by first extracting the X-ray intensity along the meridional angle Φ at $|\vec{q}|=q_{1}$, the first order lamellar diffraction peak, and fitting the resulting profile with a Gaussian distribution centered at 0. Hermans orientation function

$$\begin{array}{c} H=\frac{3<{\text{cos}}^{2}\left(\Phi \right)>-1}{2} \end{array}$$ (1)

was then used to determine the membrane orientation.

The relative electron density, *ρ*(z), was approximated by a 1-dimensional Fourier analysis [31]:

$$\begin{array}{ccc} \rho (z) & = & \frac{2}{d_{z}}\sum_{n=1}^{N}\sqrt{I_{n}q_{n}}\nu_{n}\;\text{cos}\;(\frac{2\pi nz}{d_{z}})\text{.} \end{array}$$ (2)

Here, *N* is the highest order of the lamellar peaks observed. $F\left(q_{n}\right)=\sqrt{I_{n}q_{n}}$ is the membrane’s form factor [31] and is generally a complex quantity. However, in case of centro-symmetry, the form factor becomes real and the phase problem of crystallography, therefore, simplifies to a sigmoidal problem with phase factors *v*_*n*_ = ± 1 [31]. An X-ray diffraction experiment probes the form factor at discrete values of *q*_*z*_, and a continuous function, *T*(*q*_*z*_), can be fitted to the data [32]:

$$\begin{array}{c} T\left(q_{z}\right)=\sum_{n}\sqrt{I_{n}q_{n}}\text{sinc}(\frac{1}{2}d_{z}q_{z}-\pi n). \end{array}$$ (3)

Once an analytical expression for *T*(*q*_*z*_) has been determined from fitting the experimental peak intensities, the phases *v*_*n*_ can be assessed from *T*(*q*_*z*_). The phase array *v*_*n*_ = [−1 −1 1 −1 1] was used for all samples.

The electron densities determined by Eq (2) are on a relative scale and were normalized for comparison. *ρ*(*z* = 0) was set to 0 and the electron density at the boundaries were scaled to 1.

The average size of the different lipid and peptide domains was estimated from the widths of the corresponding in-plane correlation peaks by applying Scherrer’s equation:

$$\begin{array}{c} L=\frac{0.94\lambda }{B(2\theta )\;\text{cos}(\theta )}, \end{array}$$ (4)

where λ is the wavelength of the X-ray beam, *θ* is the diffraction angle and *B*(2*θ*) is the width of the correlation peak in radians. This relation is an established method to estimate crystalline domain sizes of up to ≈100 nm in X-ray diffraction experiments. *L* corresponds to the edge size of rectangular domains in cubic lattices. We note that this method has limitations to quantitatively determine sizes of small irregular domains of a few nanometers, only. The measured values present the upper limits of the domain sizes.

The membrane interaction modulus *B* and the membrane bending modulus *κ* can be determined independently from measurements of the diffuse scattering when the membranes are hydrated close to 100% RH [33–35]. The structure factor of a well hydrated membrane is given by [33]:

$$\begin{array}{ccc} S(q_{z},q_{r}) & = & \sum_{n=-\infty }^{n=\infty }H_{z}\left(nD\right)\text{cos}\left(q_{z}nD\right)\\ & \times & \int_{0}^{\infty }rdrH_{r}\left(r\right)J_{0}\text{exp}\left(-q_{z}^{2}\delta u_{n}\left(r,\zeta ,\eta \right)/2\right), \end{array}$$ (5)

where *J*_0_ is the zero order bessel function, *H*_*r*_(*r*) and *H*_*z*_(*z*) account for the finite size of the membrane stack and *δu*_*n*_(*r*) is the height-height pair correlation function of a lipid bilayer. The definitions of all functions can be found in Lyatskaya *et al*. [33]. The bending modulus *κ* and membrane compression modulus *B* can be determined by simultaneously fitting Eq (5) to two *q*_||_-line cuts (at $q=2\frac{2\pi }{d}$ and $q=2.5\frac{2\pi }{d}$ for instance) [33, 36]. The numerical procedure for calculating *S*(*q*_*z*_, *q*_*r*_) has been described in [33]. The parameters *η* and *ξ* are the Caillé parameter [37] and the in-plane correlation length that are related to both the bending modulus *κ* and the membrane interaction modulus *B* by

$$\begin{array}{c} \eta =\frac{k_{B}Tq_{1}^{2}}{8\pi \sqrt{B\kappa }}\;and\;\xi^{4}=\frac{\kappa }{B}. \end{array}$$ (6)

Errors were determined as fit standard errors, corresponding to 95% confidence bounds, equivalent to two standard deviations, *σ*. Errors for calculated parameters, such as peak area, were then calculated by applying the proper error propagation.

### Molecular dynamics simulations

MD simulations were performed on a GPU accelerated computer workstation using GROMACS Version 5.1.4. The device was equipped with a 40 Core central processing unit (CPU, Intel(R) Xeon(R) CPU E5–2630 v4 @ 2.20GHz), 130 GB random-access memory (RAM) and three graphic processing units (GPU, 2 × NVIDIA 1080 TDI + 1 × GeForce GT 730). RBC_*cm*_ models were created using the CHARMM-GUI membrane-builder (http://charmm-gui.org/) [38, 39] and the Martini force field 2.2 [39]. Two models replicating the lipidomics of membranes from fresh and stored RBC were prepared. Each system consists of a membrane patch of ≈ 34 nm × 34 nm with about 2,500 lipid molecules on each leaflet and 37 water molecules per lipid corresponding to a well hydrated state of the membrane. The lipid composition of the membrane patch was adjusted to match the experimental lipidomic findings determined from mass spectrometry experiments as described below. Each lipid species was mapped to available models in the Martini 2.2 force field: An error coefficient was calculated for every available model lipid describing the difference in the tail length and the difference in tail saturation between the model and the experimental lipid. The Martini lipid with the smallest error value was then used for each experimental lipid respectively. The membrane’s asymmetry, *i.e*., the unequal distribution of lipids among both leaflets, was adjusted by using values for the compositional asymmetry published in previous coarse grained plasma membrane simulations [40]. For instance, from all simulated PC lipids 75% were placed in the upper and 25% were placed in the lower leaflet. Details about the exact lipid composition of each model can be found in the *Supplementary Material* in S1 Table. S1 and S2 Figs show the relative concentrations of lipid species in both membrane models. Simulating asymmetric membranes poses the risk of an an uneven area per lipid and induced curvature [41] which can impact calculated fluctuation spectrum. We thus repeated the simulations of the 30 mol% asymmetric membrane with two symmetrized membrane patches. The lipid composition from the upper and lower leaflet of the asymmetric model were taken, respectively, and used in the creation of models with symmetric leaflets.

Simulations were equilibrated for 80 ns using the NPT ensemble (constant pressure and temperature), and then run for 2 *μ*s. Only the final 1,800 ns were analyzed, after confirming the membrane had reached equilibrium by determining the area per lipid. Prior to each simulation run, the system was allowed to equilibrate for simulated 5 ns. The simulation used a 1 fs time step, a short range van der Waal cutoff of 1.1 nm and a potential-shift-verlet coulomb modifier. Periodic boundary conditions were applied to all spacial directions. Neighbor lists were updated in intervals of 20 steps. The temperature coupling was controlled by a v-rescale thermostat at a constant pressure of 1 bar using Parrinello-Rahman semi-isotropic weak coupling (*τ* = 12 ps; compressibility *β* = 3⋅10^−4^ bar^−1^). Cholesterol density maps were calculated using the gmx densmap function provided by GROMACS. Out-of-plane density profiles were calculated using the GROMACS built-in *gmx density* function. The domain size of cholesterol rich areas was determined by manually selecting 40 points on the edges of the observed clusters in the calculated density maps and measuring the distance between the points, respectively. The domain sizes of cholesterol depleted areas were determined the same way.

Fluctuation spectra for both membrane models were determined. Index files containing C1 Beads from DPGG, OPGG, FPGG, and DFGG; GL1 beads from FPMG, OPMG, DPMG and PO4 beads from POPC were created for each leaflet respectively. Trajectories for all index groups were exported between 200 ns and 2 *μ*s in steps of 4 ns from the simulation. The location of these coarse grained beads corresponds to the location of the lipid head groups in the simulated bilayer. The undulation profile at a given time step was determined by first interpolating the *z*-position of all beads for both leaflets respectively through the MATLAB built-in functin *griddata* and calculating the average undulation of the upper and lower leaflet. The 2-dimensional spectrum was then determined using built-in MATLAB functions. The scaling of the spectrum was verified using the program provided by the authors of [42].

The spectrum is governed by a *q*^4^ dependency according to the Helfrich–Canham (HC) theory. The bending modulus can be thus determined by fitting the lower *q*-regime (*q* < 0.1 Å^−1^) to

$$\begin{array}{c} {\left\langle |h\left(q\right)\right|}^{2}\rangle =\frac{k_{B}T}{\kappa q^{4}} \end{array}$$ (7)

### Lipidomics analysis

#### Lipidomics

Samples were resolved as described [43], over an ACQUITY HSS T3 column (2.1 × 150 mm, 1.8 *μ*m particle size (Waters, MA, USA) using an aqueous phase (A) of 25% acetonitrile and 5 mM ammonium acetate and a mobile phase (B) of 50% isopropanol, 45% acetonitrile and 5 mM ammonium acetate. Samples were eluted from the column using either the solvent gradient: 0–1 min 25% B and 0.3 ml/min; 1–2 min 25–50% B and 0.3 ml/min, 2–8 min 50–90% B and 0.3 ml/min, 8–10 min 90–99% B and 0.3 ml/min, 10–14 min hold at 99% B and 0.3 ml/min, 14–14.1 min 99–25% B and 0.3 ml/min, 14.1–16.9 min hold at 25% B and 0.4 ml/min, 16.9–17 min hold at 25% B and resume flow of 0.3 ml/min. Isocratic elution of 5% B flowed at 250 *μ*l/min and 25°C or a gradient from 0- 5% B over 0.5 min; 5–95% B over 0.6 min, hold at 95% B for 1.65 min; 95–5% B over 0.25 min; hold at 5% B for 2 min, flowed at 450 *μ*l/min and 35°C [44]. The Q Exactive mass spectrometer (Thermo Fisher Scientific, San Jose, CA, USA) was operated independently in positive or negative ion mode, scanning in Full MS mode (2 *μ*scans) from 150 to 1500 m/z at 70,000 resolution, with 4 kV spray voltage, 45 sheath gas, 15 auxiliary gas.

#### MS2 analyses for untargeted lipidomics

For untargeted lipidomics, dd-MS2 was performed at 17,500 resolution, AGC target = 1 ⋅ 10^5^, maximum IT = 50 ms, and stepped NCE of 25, 35 for positive mode, and 20, 24, and 28 for negative mode, as described in Stefanoni *et al*. [45] and applied to similar samples (*i.e*., stored RBCs) in D’Alessandro *et al*. [46].

#### Quality control and data processing

Calibration was performed prior to analysis using the PierceTM Positive and Negative Ion Calibration Solutions (Thermo Fisher Scientific). Acquired data was then converted from.raw to.mzXML file format using Mass Matrix (Cleveland, OH, USA). Samples were analyzed in randomized order with a technical mixture (generated by mixing 5 *μ*l of all samples tested in this study) injected every 10 runs to qualify instrument performance. This technical mixture was also injected three times per polarity mode and analyzed with the parameters above, except CID fragmentation was included for unknown compound identification (10 ppm error for both positive and negative ion mode searches for intact mass, 50 ppm error tolerance for fragments in MS2 analyses—further details about the database searched below).

#### Metabolite assignment and relative quantitation

Metabolite assignments, isotopologue distributions, and correction for expected natural abundances of deuterium, ^13^C, and ^15^N isotopes were performed using MAVEN (Princeton, NJ, USA) [47], against an in house library of deuterated lipid standards (SPLASH LIPIDOMIX Mass Spec Standard, Avanti Lipids) and in house libraries of 3,000 unlabeled (MSMLS, IROATech, Bolton, MA, USA; IroaTech; product A2574 by ApexBio; standard compounds for central carbon and nitrogen pathways from SIGMA Aldrich, St Louis, MO, USA) and labeled standards (see below for the latter). Untargeted lipidomics analyses were performed with the software LipidSearch (Thermo Fisher, Bremen, Germany). Results from lipidsearch were exported as a library and additional discovery mode analyses were performed with standard workflows using Compound Discoverer 2.1 SP1 (Thermo Fisher Scientific, San Jose, CA). From these analyses, metabolite IDs or unique chemical formulae were determined from high-resolution accurate intact mass, isotopic patterns, identification of eventual adducts (e.g., Na^+^ or K^+^, etc.) and MS2 fragmentation spectra against the KEGG pathway, HMDB, ChEBI, and ChEMBL databases.

## Results

### Structure of the RBC_*cm*_ from X-ray diffraction and X-ray diffuse scattering

**Fig 1:**
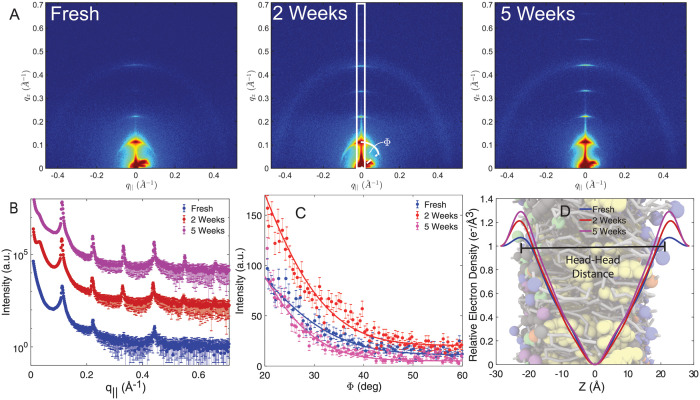
**A** Two-dimensional X-ray intensity maps measured at 88% RH for a fresh RBC_*cm*_ sample and RBC collected from RC that was stored for 2 and 5 weeks respectively. Up to 6 orders of lamellar peaks were observed. **B** Specular reflectivity as determined from the integrated intensity marked as white rectangle. **C** X-ray intensity determined along the meridional angle Φ as indicated by the white line in **A**. The degree of orientation was determined by fitting Hermans orientation function. **D** Relative electron density profile determined from a 1-dimensional Fourier analysis. Maxima around |*z*| = 20 Å indicate the location of the electron-rich head-groups of the membranes while the electron density is reduced in the bilayer center.

Two-dimensional X-ray intensity maps for membranes from fresh RBC, 2 weeks and 5 weeks old RC, all measured at 88% RH, are depicted in Fig 1A. Structural features are typically enhanced at this slightly reduced hydration. Series of pronounced lamellar peaks were observed in all samples indicating a lamellar organization of the membranes. The maximal observed order of lamellar peaks was found to be 4 for a fresh RBC_*cm*_s, and up to 6 in case of samples prepared from stored RBC. *q*_1_ will hereafter refer to the position of the first order lamellar peak. The specular reflectivity was analyzed by first integrating the 2-dimensional X-ray intensity map along the marked rectangle. The resulting line-cuts are shown in Fig 1B. The lamellar spacing *d*_*z*_ was determined from the peak positions and Bragg’s law, *d*_*z*_ = 2*π*/*q*_*z*_, as listed in Table 1. Fig 1C shows the X-ray intensity profile along the meridional angle Φ. The degree of order in the membrane stack was determined by fitting Hermans orientation function.

**Table 1:** Structural parameters and membrane bending modulus *κ* determined from XRD and XDS experiments.

	Fresh	2 Weeks	5 Weeks
Lamellar spacing *d* (Å)	55.4 ± 0.5	56.9 ± 0.2	57.3 ± 0.1
HH-distance *d*_*HH*_ (Å)	43 ± 1	46 ± 1	45 ± 1
Water-layer thickness (Å)	11.9 ± 1	10.9 ± 1	12.3 ± 1
Membrane order parameter (%)	88.4 ± 0.7	89.9 ± 0.5	91.5 ± 0.5
Ratio *l*_*d*_:*l*_*o*_	60:40	59:41	54: 46
Lipid tail distance *a* (Å)			
*l*_*d*_ domains	5.39 ± 0.03	5.39 ± 0.004	5.413 ± 0.005
*l*_*o*_ domains	4.69 ± 0.27	4.20 ± 0.01	4.22 ± 0.01
Protein spacing	10.88 ± 0.22	10.54 ± 0.07	10.33 ± 0.06
Area per lipid tail *A*_*T*_ (Å^2^)			
*l*_*d*_ domains	25.18 ± 0.13	25.173 ± 0.008	25.383 ± 0.008
*l*_*o*_ domains	19.04 ± 1.10	15.29 ± 0.02	15.44 ± 0.03
Domain size *ζ* (Å)			
*l*_*d*_	29 ± 2	24.6 ± 0.31	22.7 ± 0.1
*l*_*o*_	16 ± 3	14.2 ± 0.3	12.1 ± 0.4
Bending modulus *κ* (k_B_T)	1.9 ± 0.2	4.6 ± 0.4	5.3 ± 0.4

Out-of-plane electron density profiles were determined by a 1-dimensional Fourier analysis and are shown in Fig 1D. The electron density is plotted on a relative scale. The maximal electron density was observed around |*z*| = 20 Å indicating the location of the electron-rich head-groups of the membranes; it reaches a minimum in the center of the membrane. While the differences in the electron density between fresh RBCs and stored RBCs were small within the membranes, the electron density in the head-groups of the 2 and 5 weeks sample was observed to be increased by 10% and 20%, respectively, as compared to a fresh RBC sample.

The distance between the head-group peaks was defined as membrane thickness *d*_*HH*_ and is listed Table 1. The difference between the *d*_*z*_-spacing and the membrane thickness corresponds to the thickness of the water layer between neighboring membranes (also listed in Table 1).

**Fig 2:**
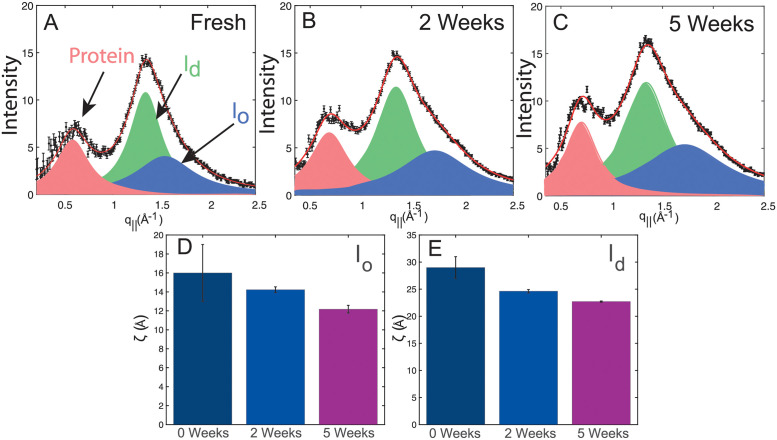
**A—C** Background corrected in-plane X-ray intensity profiles. Three peaks resulting from liquid ordered *l*_*o*_ and liquid disordered *l*_*d*_ lipid domains, as well as *α*-helical protein structures were observed and fits are shown in blue, green and red respectively. The domain sizes were determined as described in Materials & methods and are expressed as the edge length of the domain. They are plotted in **D** and **E**.

Fig 2 shows the in-plane diffraction signal from 2 and 5 weeks stored RBC_*cm*_s. Peaks at *q*_||_ = 0.7 Å^−1^, 1.3 Å^−1^ and 1.7 Å^−1^ were observed. The blue and green signals are the result of a hexagonal packing of the liquid disordered (*l*_*d*_) and liquid ordered (*l*_*o*_) lipid tails in the hydrophobic membrane core (planar group p6) [29]. A third peak shown in red was assigned to coiled-coil *α*-helical peptides [29]. The distance between two acyl tails was determined using $a=4\pi /(\sqrt{3}q_{\left|\right|})$, where q_||_ is the position of the corresponding correlation peak. The area per lipid chain is obtained to $A_{T}=\left(\sqrt{3}/2\right)a^{2}$. Values for the area per lipid chain in *l*_*o*_ and *l*_*d*_ domains are listed in Table 1. The tail distance in *l*_*o*_ domain was found to slightly decrease during storage, while changes in the tail distance of *l*_*d*_ domains were within statistical errors. However, the fraction of *l*_*d*_ domains was found to monotonically decrease by 6% in favor of *l*_*o*_ domains. The sizes *ζ* of both lipid domains are plotted in Fig 2C and 2D and were found to monotonically decrease.

**Fig 3:**
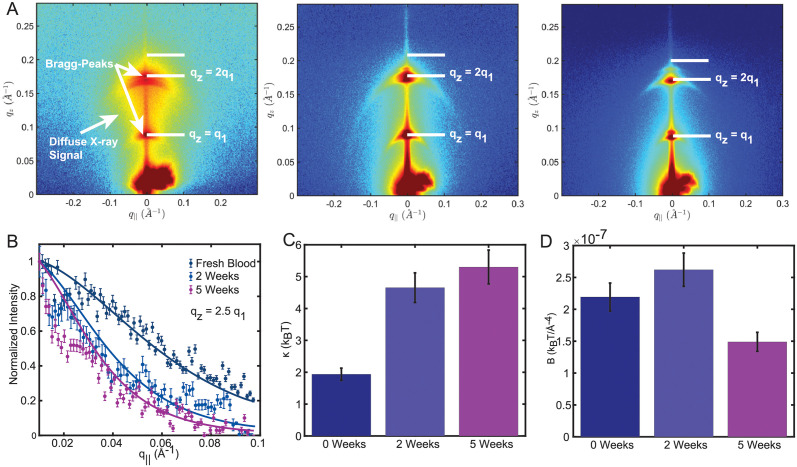
**A** Two-dimensional X-ray intensity maps measured at 99.9% RH. A diffuse cloud of X-ray signal was observed as the result of out-of-plane fluctuations. Line-cuts at *q*_*z*_ = 2.5 ⋅ *q*_1_ are depicted in **B**. The bending modulus *κ* and membrane interaction modulus *B* were determined by fitting *S*(*q*) to the data, and are visualized as bar graph in **C** and **D**.

Diffuse scattering was measured in Fig 3A, showing 2-dimensional X-ray intensity maps measured at 99.9% RH. Only two orders of lamellar peaks were observed as the result of increased fluctuations at this high hydration. Importantly, a diffuse cloud of X-ray signal was detected around the peaks resulting from membrane height fluctuations. Line-cuts at *q*_*z*_ = 2 ⋅ *q*_1_ and *q*_*z*_ = 2.5 ⋅ *q*_1_ were taken, and are depicted in Fig 3B and S4 Fig in the *Supplementary Material*. The membranes’ bending modulus *κ*, and compressibility modulus *B* were determined by fitting the calculated structure factor *S*(*q*) (in Eq (5)) to the diffuse profiles. Bending moduli of *κ* = 4.6 k_B_T and *κ* = 5.3 k_B_T were determined for membranes extracted from RBCs after 2 and 5 weeks of storage, indicating a 2.8× fold increase as compared to fresh RBC (*κ* = 1.9 k_B_T).

The results of the structural analysis (in Table 1) can be summarized as follows: lamellar spacing and membrane thickness increase during storage and the membranes become stiffer as the order parameter increases. At the same time the fraction of *l*_*d*_ domains decreased from 60% to 54% while the fraction of *l*_*o*_ lipids increased from 40–46%. The size of those domains reduced from 29 Å to about 23 Å which suggests a higher dispersion of these domains in the membrane. The bending rigidity *κ* monotonically increased with storage time from 1.9 to 5.3 k_B_T.

### Membrane lipidomics

Lipidomics of RBC_*cm*_ was measured and analyzed with respect to the abundance of lipid species, tail length and degree of tail saturation. S1A and S2A Figs in the *Supplementary Material* show the abundance of Phosphatidylcholine, PC; Ceramide, CER; Monoglucosyl lipids, MG; Diacylglycerol lipids, DG; Fatty acids, FA; Sphingomyelin, SM; Phosphatidylethanolamine, PE; Phosphatidylserine, PS; Phosphatidylglycerol, PG; Phosphatidic acid, PA; Phosphatidylinositol, PI for a fresh RBC sample and a sample after 42 days. S3A and S3B Fig compare the differences in tail saturation and tail length for a fresh and 42 day old RBC_*cm*_. It was found that the difference between the samples was small and in the order of a few percent, only. The abundance of tails with a length < 16 CH_2_ groups was found to be decrease by 5% in favor of shorter tails with a length of 8 and 12 CH_2_ groups. At the same time the tails were found to be more unsaturated. While fatty acids accounted for less than 1% of the RBC_*cm*_ in the fresh sample, they contributed ≈5% to the RBC_*cm*_’s lipidomics in a 42 day old sample (S3C Fig). While this analysis method provides detailed insight into the RBC_*cm*_’s lipid composition, the information about cholesterol concentrations is limited. While [27] reported that cholesterol makes up one third of the membrane in fresh cells and half of the RBC_*cm*_ after 42 days of storage [27], others assume that cholesterol typically makes 50 mol% of the lipid content, and that the concentration does not change during storage [28].

### Molecular dynamics simulations

Two coarse grained membrane models mimicking a fresh RBC_*cm*_ and a RBC_*cm*_ after 42 days of storage were created. To mimic the largest potential change in composition, lipidomics from fresh RBC and RBC after 42 days of storage were used with cholesterol concentrations of 30 mol% and 50 mol%, respectively.

**Fig 4:**
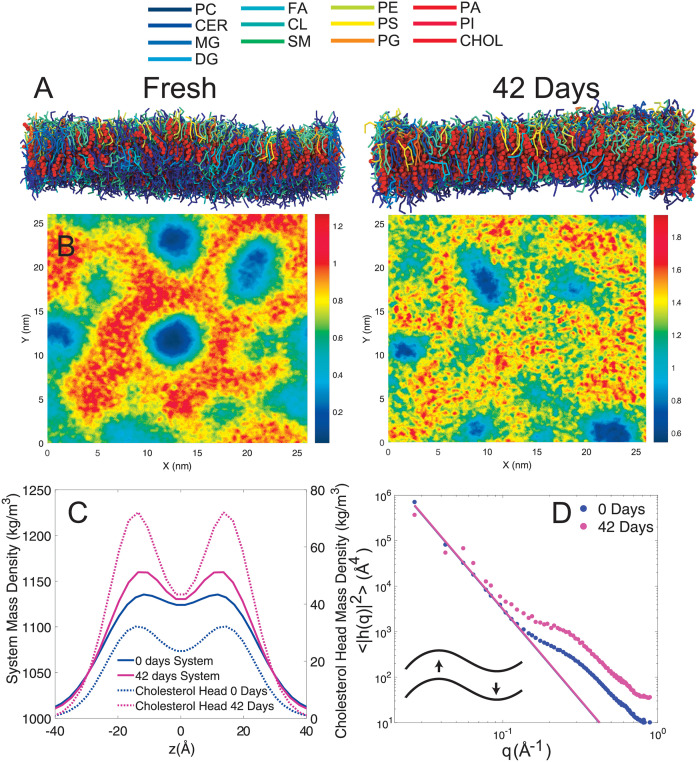
**A** 3-dimensional render of a simulated RBC_*cm*_ patch after 2 *μ*s. Lipid molecules are displayed as rods symbolizing molecular bonds. Cholesterol is depicted as red spheres. Each lipid species (Phosphatidylcholine, PC; Ceramide, CER; Monoglucosyl lipids, MG; Diacylglycerol lipids, DG; Fatty acids, FA; Sphingomyelin, SM; Phosphatidylethanolamine, PE; Phosphatidylserine, PS; Phosphatidylglycerol, PG; Phosphatidic acid, PA; Phosphatidylinositol, PI) are represented by different colors indicated in the legend. **B** Cholesterol density maps averaged over the last 800 ns of the simulation. Red indicates cholesterol rich areas while blue represents cholesterol depletion. **C** Mass density profiles averaged over the last 800 ns of the simulation for the entire membrane patch (System) and the cholesterol head groups. **D** Fluctuation spectrum of the 42 day old membrane patch. The bending modulus *κ* was determined by fitting Helfrich–Canham (HC) theory.

Fig 4A shows 3-dimensional renders created after 2 *μ*s of simulations, where lipid molecules are represented by rods and cholesterol molecules are displayed as red spheres. Cholesterol density maps were created and are shown in Fig 4B. A patchy structure is apparent, where cholesterol rich areas are shown in red; blue areas indicate cholesterol depletion. Domain sizes were measured with values of ≈50 Å for fresh, and 40 Å in 5 weeks old membranes (listed in Table 2). Fig 4C shows the mass density profiles of the overall membrane patch (solid lines) and the cholesterol OH-Group (dotted lines) that were determined from both simulations. A mass density of ≈1,000 kg/m^3^ was observed in the water layer surrounding the membrane. The density was significantly increased within the bilayer and peaks around the membrane’s head-group region of the membrane. This head-group mass density was found to be increased by up to 25 kg/m^3^ in the 42 day old membrane mimic as compared to the fresh membrane. The increase was 5 kg/m^3^ in the bilayer center only. The cholesterol’s head-group density was observed to increase by a factor of 2.3 from a maximum of 31 kg/m^3^ in the fresh membrane mimic to a maximum of 71 kg/m^3^ in the 42 days membrane patch.

**Table 2:** Structural parameters and bending modulus *κ* determined from MD simulations.

	fresh	5 weeks
Domain Size *ζ* (Å)		
*l*_*d*_	52 ± 16	40 ± 17
*l*_*o*_	42 ± 17	30 ± 1
Bending Modulus *κ* (k_B_T)	3.2 ± 0.1	5.3 ± 1.5

The fluctuation spectrum is shown in Fig 4D. It follows a *q*^4^ dependency in the low-*q* regime (*q* < 0.1 Å^−1^), as predicted by the Helfrich–Canham (HC) theory (Eq (7)), which describes membrane undulations on length scales much larger than the membrane thickness [42, 48]. The spectrum deviates from the *q*^4^ dependency for *q* > 0.1 Å^−1^. Fits of Eq (7) for values of *q* < 0.1 Å^−1^ are displayed as red solid line. There was an increase in bending modulus from *κ* = 3.2 ± 0.1 k_B_T for fresh membranes, to *κ* = 5.3 ± 1.5 k_B_T for the 5 week old membrane patch.

Due to a random stacking of membranes in the solid supported RBC_*cm*_ samples, the XRD experiment is not sensitive to a potential asymmetry of the bilayers. The simulation containing 30 mol% cholesterol was thus repeated with a symmetric lower and upper leaflet respectively. The fluctuation spectrum of both simulations is shown in S5 Fig (*Supplementary Material*). The bending modulus was measured to be 4.12 ± 1.36 k_B_T (symmetric upper leaflet) and 3.18 ± 0.84 k_B_T (symmetric lower leaflet) and thus agrees with the asymmetric membrane patch within statistical errors.

The simulations thus indicate a decrease in domain size, a decrease in the fraction of *l*_*d*_ domains and an increase in the bending modulus, *κ*, of the membranes as function of storage time, in agreement with the experimental findings.

## Discussion

As a general note, the observed changes in the hemoglobin depleted RBC_*cm*_ structure during storage were small and require high-resolution techniques to be resolved. The majority of lipid species (PC, PE, PA, PI, PS, PG, MG and DG) were found to change only slightly, in the order of ≈1%. Only the concentration of fatty acid was found to increase by ≈5%. It has been previously reported that the degree of fatty acid unsaturation increased in stored RBCs as a function of oxidant stress and pyruvate/lactate ratios, perhaps as a result of residual fatty acid desaturase activity in the mature RBC or moonlighting function of other enzymes sensitive to NADH/NAD^+^ ratios [49].

A significant change was reported in the membranes’ cholesterol content by [27], who observed an increase from 30 mol% to 50 mol%, which was speculated to be a result of lipid loss [26]. The rigid cholesterol molecule is widely known to form patches with increased lipid tail order within the membrane [50–56], so-called liquid-ordered *l*_*o*_ domains. Our experimental results show that the fraction of liquid disordered *l*_*d*_ domains decreases by 6% in favor of those liquid ordered *l*_*o*_ domains. At the same time the sizes of both domains were found to decrease. The same changes were observed for the cholesterol rich patches in the MD simulations, suggesting that the experimental observations are the effect of higher cholesterol concentrations resulting in a splitting and dispersion of the domains in stored RBC_*cm*_.

We note a discrepancy in the values of the domain sizes determined in XRD experiments and MD simulations ($\zeta_{l_{d}}=29\;Å$ and $\zeta_{l_{o}}=16\;Å$ for the fresh RBC_*cm*_ in XRD experiments *vs*. $\zeta_{l_{d}}=52\;Å$ and $\zeta_{l_{o}}=42\;Å$ for the fresh RBC_*cm*_ in MD simulations). Scherrer’s equation (Eq 4) was developed for the study of crystalline structures and measures the domain size from the width and position of in-plane correlation peaks. The equation generally determines the edge size of quadratic domains in a presumably cubic lattice. However, lipid domains have a rather irregular shape, as it is apparent from the simulation. A maybe more appropriate comparison between both results is thus given by diagonal elements in the quadratic domains which increases the experimental sizes by a factor of $\sqrt{2}$, which brings experimental and computational findings in good agreement.

The *l*_*o*_ domains have a decreased area per lipid tail (*A*_*T*_ = 19 Å^2^ in *l*_*o*_ domains *vs*.*A*_*T*_ = 25 Å^2^ in *l*_*d*_ domains) which was found to decrease slightly (*A*_*T*_ = 19 Å^2^ in fresh RBC_*cm*_ *vs*.*A*_*T*_ = 15 Å^2^ in stored RBC_*cm*_) in the stored samples. Importantly, this denser packing of molecules, together with the measured larger fractions of *l*_*o*_ domains, well explains the increase in the system’s mass and electron density observed in simulations and experiments. The increased fraction of these patches thus agrees with the observed age dependent increased electron and mass density in both experiments and simulations. The measured increased HH-distance was found to be small (2 Å). However, our lipidomic findings report negligible changes in the tail length of the lipid molecules and the increased membrane thickness can consequently be understood as the result of straightened lipid tails in cholesterol rich domains. This is also supported by the 6% increase of membrane order parameter in the XRD experiments.

Cholesterol is known to reduce the non-active oxygen transport across lipid bilayers [21, 57, 58]. The denser packing of lipid tails around cholesterol molecules presents a physical barrier [58] and oxygen more likely transits the membrane at the boundaries between *l*_*o*_ and *l*_*d*_ domains [21]. The observed larger fraction of *l*_*o*_ domains with an denser lipid packing in stored RBC_*cm*_ suggests that there is less space available for oxygen to permeate the membrane. We thus speculate that the changes in the RBC_*cm*_’s domain landscape may influence the passive transport of oxygen across the membrane, which is of particular importance for this cell species.

A significant increase (2.8×) in the membrane’s bending modulus was observed in XDS experiments. These observations are consistent with the RBC_*cm*_ becoming stiffer during storage. It further agrees well with previous studies reporting a decreased deformability of stored RBCs [15–19]. It is well known that RBCs have a composite outer “shell” formed by a cytoplasmic membrane (RBC_*cm*_) tethered to a spectrin network. We argue that our results measure the bending modulus of solely the RBC_*cm*_ in the absence of the spectrin network. Spectrin filaments were no longer detectable using our preparation protocol [29] by fluorescent microscopy following sonication with subsequent centrifugation of RBC ghosts. In addition, the *d*_*z*_-spacing in XRD experiments together with electron density profiles are inconsistent with the presence of spectrin structures between membranes in the solid supported stack.

The increase in the bending modulus observed in the experiments is thus related to a stiffening of the RBC_*cm*_. An obvious explanation for this increase may be an increased cholesterol concentration in stored membranes. To address this question, MD simulations were performed in the absence of proteins to explicitly probe the influence of membrane composition and in particular the effect of cholesterol. However, the simulations show a slight increase in the membrane’s bending modulus, only, substantially smaller than in the XDS experiments. This difference becomes even smaller when considering results on membrane patches with symmetric upper and lower leaflet. We conclude that the significant increase in cholesterol concentration is obviously not directly linked to increased stiffness in biological membranes. Cholesterol’s rigid molecular structure contrasts the flexible nature of fatty acyl tails and is known to increase the membrane’s bending modulus in fully saturated model membranes [59, 60]. However, this effect is substantially reduced for mono-unsaturated bilayer and vanishes for 1,2-dioleoyl-sn-glycero-3-phosphocholine (DOPC) membranes [59, 60]. Little is known about the effect of cholesterol on the bending rigidity of multi-component lipid bilayers. Only ≈1/3 of the lipids within the RBC_*cm*_ were found to be fully saturated and the findings are thus in-line with previous experiments on synthetic membranes and re-emphasize the small effect of cholesterol on the membrane’s bending rigidity when there are unsaturated molecules present. The change in *κ* is more likely related to the effect of integral membrane proteins, as has been speculated before [42, 61–63]

Blood bags are primarily composed of polyvinyl chloride (PVC) compounded with 30–40% *wt* di(2-ethylhexyl) phthalate (DEHP) [64], a plasticizer used to improve the bags’ flexibility and durability. Due to its lipophilic structure, DEHP is known to migrate from the PVC polymer matrix into packed red blood cells (RBC) [65, 66]. 15 to 624.2 *μ*g/ml of DEHP were detected in PVC blood bags after 20 days of storage [67, 68] and 7.4 to 36.1 *μ*g/ml of DEHP were found in irradiated RBC concentrate products [67]. The partitioning of DEHP in RBC_*cm*_s has long been suspected to change membrane properties [68, 69] and contribute to the changes observed during storage. A recent study in model lipid bilayers indeed reported that DEHP can increase membrane width and area per lipid, and the deuterium order parameter, however, decrease membrane orientation, indicating the formation of thicker, stiffer membranes with increased local curvature [70]. Concentrations of DEHP in this paper were elevated, of up to 10 mol% of the lipid concentration, to emphasize the potential effects of DEHP. Even though the presence of DEHP could potentially explain the observed increased stiffness in our experiments, we could not find clear evidence for the presence of DEHP molecules in the electron density of RBC_*cm*_s in our X-ray diffraction results. The upper bound of experimentally reported DEHP concentrations in blood bags is ≈ 600 *μ*g/ml [68], which corresponds to molar concentrations of less than 0.2 mol%, significantly smaller than what was used in [70]. While we can not rule out that the effects of DEHP were too small to be detected in this study, we speculate that the changes in the membranes’ structural parameters would be even smaller than the subtle effects reported by Bider *et al*.

## Conclusion

The molecular structure of RBC_*cm*_ was determined from fresh RBCs and RBCs that were stored for 2 and 5 weeks in commercial blood bags respectively. We provide direct experimental evidence for an increased fraction of liquid ordered lipid domains within the bilayer. This is consistent with an observed increase in the membrane thickness, membrane order parameter and head-group density as a result of straightened lipid tails in these cholesterol rich domains. The domain size of *l*_*o*_ and *l*_*d*_ lipid domains was found to decrease with storage time. X-ray diffuse scattering experiments revealed a significant increase (2.8×) of the membrane’s bending modulus *κ*. A smaller increase (factor 1.6) from *κ* = 3.2 k_B_T to *κ* = 5.3 k_B_T was observed in MD simulations which did not contain proteins. These results suggest a small impact of the altered membrane composition on the RBC_*cm*_’s bending rigidity. The discrepancy between experiment and simulation is likely related to the effect of integral membrane proteins.

## Supporting information

S1 Table Lipid composition of RBC_*cm*_s from fresh RBC and stored RBC as determined from mass spectrometry and corresponding mapped coarse grained molecular dynamics models. (XLSX)

S1 Fig A Experimentally determined composition of red blood cell membrane as reported by [45]. B Lipidomics of the implemented coarse grained MD simulation model. The asymmetry of the membrane was created by distributing lipids between both leaflets according to experimental findings by [40]. C Comparison of the degree of tail saturation in the experimental and model membrane. D Comparison between the lipid tail length of the experimental and model membrane.
(EPS)

S2 Fig A Experimentally determined composition of red blood cell membrane as reported by [45]. B Lipidomics of the implemented coarse grained MD simulation model. The asymmetry of the membrane was created by distributing lipids between both leaflets according to experimental findings by [40]. C Comparison of the degree of tail saturation in the experimental and model membrane. D Comparison between the lipid tail length of the experimental and model membrane.
(EPS)

S3 Fig Comparison of the experimentally determined distribution of lipid tail lengths (A) and degrees of tail saturation (B). C Concentration differences of fatty acids. D The cholesterol concentration in fresh RBC and stored RC as determined by [27].
(EPS)

S4 Fig Line cuts at *q*_*z*_ = 2*q*_1_ and corresponding fits of *S*(*q*) (Eq (5)). (EPS)

S5 Fig Fluctuation spectra determined from simulations of a symmetrisized versions of the asymmetric membrane patch (30 mol%). Bending moduli of *κ* = 4.1 ± 1k_B_T and *κ* = 3.1 ± 0.8k_B_T were determined for membranes with a symmetric upper and lower leaflet respectively.
(EPS)
